# Mineralocorticoid receptor antagonists in heart failure: a systematic review and meta-analysis

**DOI:** 10.3389/fcvm.2025.1667236

**Published:** 2025-09-01

**Authors:** Yue Zhang, Yin-Chao Bao, Li-Xia Wang, Hong-Juan Zhao, Jing Sun, Lin Sun, Wei Duan, Ming Du, Lei-Jun Wang, Qin-Yan An, Wen-Ze Yang

**Affiliations:** ^1^ Tongji University School of Medicine, Shanghai, China; ^2^ Department of General Practice, Datuan Community Health Service Center, Pudong New Area, Shanghai, China; ^3^ Department of Respiratory, Sijing Hospital of Songjiang District, Shanghai, China; ^4^ Department of Cardiology, Sijing Hospital of Songjiang District, Shanghai, China; ^5^ Department of Cardiology, Jiuquan Hospital Affiliated to Shanghai First People’s Hospital, Jiuquan City, Gansu, China

**Keywords:** mineralocorticoid receptor antagonists, heart failure and reduced ejection fraction, HF and mildly reduced ejection fraction, HF with preserved ejection fraction, hyperkalemia

## Abstract

**Background:**

Mineralocorticoid receptor over-activation drives maladaptive myocardial fibrosis, vascular inflammation and renal sodium retention across the entire spectrum of left ventricular ejection fraction (LVEF). While steroidal MRAs have convincingly reduced hospitalizations and mortality in patients with heart failure and reduced EF (HFrEF), evidence remains fragmented for heart failure with mildly reduced (HFmrEF) or preserved EF (HFpEF), and no head-to-head data distinguish steroidal from non-steroidal agents. This study aimed to evaluate the effect of MRAs in patients with HF across the range of ejection fraction.

**Methods:**

Searched PubMed, Web of Science, Wanfang and Cochrane library (1 Jan 1987–10 Sep 2024) for randomized clinical trials (RCT) assessing MRAs (finerenone, spironolactone, eplerenone) in HFpEF, HFmrEF or HF. The primary endpoint was composite cardiovascular (CV) outcomes. Secondary endpoints included CV mortality, overall HF exacerbation events, safety, and adverse events. A meta-analysis was conducted using hazard ratios (HR), confidence intervals (CI), and relative risks (RR) to synthesize the findings.

**Results:**

In the analysis of nine RCTs, MRAs were associated with a 23% reduction in CV composite outcomes (RR: 0.77, 95% CI: 0.72–0.83, *P* < 0.00001), a 23% reduction in HF hospitalization risk (HR: 0.77, 95% CI: 0.70–0.84, *P* < 0.00001), and a 22% reduction in all-cause mortality (HR: 0.78, 95% CI: 0.72–0.85, *P* < 0.00001) in HFrEF patients, compared to a 17% reduction in CV composite events (HR: 0.85, 95% CI: 0.78–0.93, *P* = 0.0004), a 20% reduction in HF hospitalization risk (HR: 0.80, 95% CI: 0.73–0.89, *P* < 0.00001), and an 8% reduction in all-cause mortality (HR: 0.91, 95% CI: 0.85–0.99, *P* = 0.02) in HFmrEF/HFpEF patients. However, CV mortality was not significantly reduced in HFmrEF/HFpEF patients (HR: 0.92, 95% CI: 0.82–1.02, *P* = 0.13), but was reduced by 23% in HFrEF patients (HR: 0.77, 95% CI: 0.70–0.83, *P* < 0.00001). The incidence of any serious adverse events was similar between the MRA and placebo groups. The incidence of hyperkalemia was significantly higher in the MRA group (RR: 2.19, 95% CI: 1.97–2.43, *P* < 0.00001).

**Conclusions:**

MRAs should be considered for patients with HFrEF due to their substantial benefits. In HFmrEF or HFpEF, MRAs may confer benefit, though the effect is modest and hyperkalemia risk is higher, mandating close potassium monitoring.

**Systematic Review Registration:**

PROSPERO CRD42022304966.

## Introduction

Mineralocorticoid receptor antagonists (MRAs) are a cornerstone in the management of heart failure (HF), particularly in patients with heart failure with reduced ejection fraction (HFrEF) (1). In 2023, the European Society of Cardiology (ESC) issued a focused update to its 2021 guidelines on acute and chronic heart failure, adding a class IIb recommendation for MRAs in patients with mildly reduced (HFmrEF) or preserved (HFpEF) ejection fraction. Crucially, the document declines to endorse any specific MRA for the HFpEF cohort, underscoring prevailing clinical caution in drug selection for this phenotype (2). In September 2024, FINEARTS showed finerenone significantly reduced the composite of worsening HF events and cardiovascular (CV) death, providing robust evidence for its use in HFmrEF and HFpEF (3).

Spironolactone and eplerenone are recommended for HFrEF to reduce mortality and hospitalization. They have shown significant efficacy in reducing CV death and HF hospitalizations in HFrEF patients (4, 5). Finerenone, a newer nonsteroidal MRA, has demonstrated efficacy in reducing CV mortality and hospitalizations in HFpEF and HFmrEF, with a lower risk of hyperkalemia compared to steroidal MRAs (6). Both steroidal and nonsteroidal MRAs are included in HF management guidelines, but their use is often limited by the risk of hyperkalemia and renal impairment (7).

Most studies have concentrated on HFrEF, leaving a gap in data for HFmrEF and HFpEF. Currently, robust evidence supports steroidal MRAs like spironolactone and eplerenone for HFrEF management, though they carry risks of hyperkalemia and sex hormone-related side effects. Non-steroidal MRA finerenone offers higher receptor selectivity and a potentially safer profile, with its heart failure evidence mainly from diabetic nephropathy trials. In July 2025, finerenone was FDA-approved for HFmrEF and HFpEF, and Japanese guidelines have a Class IIa recommendation for its use in these conditions (8). The efficacy of MRAs in these subtypes remains less clear and inconsistent across studies (4, 9). There is a lack of direct comparative studies between steroidal and nonsteroidal MRAs, particularly in patients with renal insufficiency and other comorbidities (10). The risk of hyperkalemia and renal function deterioration is a significant concern, especially in patients with chronic kidney disease (CKD), which limits the broader application of MRAs (9, 11).

We conducted a comprehensive meta-analysis to evaluate the efficacy and safety of MRAs in HF patients across the EF spectrum, including HFrEF, HFmrEF, and HFpEF.

## Methods

### Study design

This systematic review and meta-analysis was conducted to evaluate the efficacy and safety of steroidal and non-steroidal MRAs in patients with HFrEF, HFmrEF or HFpEF. The study adhered to a predefined protocol (PROSPERO: CRD 42022304966) and followed the guidelines outlined in the PRISMA statement for conducting this meta-analysis (Table 1). This meta-analysis focuses on symptomatic HF patients, including HFrEF: HF with LVEF ≤ 40%; HFmrEF: HF with LVEF 41%–49%; HFpEF: HF with LVEF ≥ 50% (12).

### Search strategy, selection criteria, and data extraction

A systematic search was performed in PubMed, Web of Science, WANGFANG and Cochrane library from January 1, 1987 through September 10, 2024. The search strategy incorporated the terms “finerenone”, “spironolactone”, “eplerenone”, “HFpEF”, “HFmrEF,” and “heart failure” to identify relevant studies.

Eligible for this analysis were randomized clinical trials (RCT) designed to appraise the therapeutic efficacy of pharmacological agents recommended for the management of HFrEF, HFmrEF and HFpEF. The pharmacotherapies of interest in this study comprised spironolactone, eplerenone, and finerenone. We confined our search to trials involving adult subjects, 18 years of age or older, diagnosed with HFmrEF or HFpEF, as defined by the EF surpassing 40%. The stringent criteria for participant selection were implemented to guarantee the pertinence of our study cohort and to facilitate the extrapolation of our results to the broader demographic of adults grappling with these cardiac affections. Patients were excluded if they had an estimated glomerular filtration rate (eGFR) < 25 ml/min/1.73 m^2^, serum potassium >5.0 mmol/L, recent MRA use within 30 days, or a history of specific cardiomyopathies (e.g., dilated, peripartum, chemotherapy-induced, or amyloidosis cardiomyopathies), as well as HF symptoms from non-cardiac causes.

Two investigators (Y.Z. and Y-C.B.) conducted a thorough screening of titles and abstracts from the identified citations to ascertain eligibility. Subsequently, full texts of selected citations were independently reviewed by the same two investigators. Any discrepancies were resolved by consensus. Two authors extracted data from studies, cross-checking for accuracy.

### Outcomes

The primary endpoint was a composite measure of CV mortality and HF events, encompassing CV death, hospitalizations due to HF, resuscitated cardiac arrests, initial hospitalizations for HF, and all episodes of worsening HF. The secondary outcomes included all-cause mortality, CV death, and hospitalization for HF. Safety outcomes were examined in patients who received at least one dose of randomly assigned treatment. The safety outcomes included serum creatinine ≥2.5 mg/dl, a decrease of more than 20% in eGFR, hypokalemia or hyperkalemia (serum potassium <3.5 mmol/L or >5.5 mmol/L), and systolic blood pressure <100 mm Hg.

### Methodological quality

Risk of bias was evaluated in accordance with the Cochrane Collaboration's tool for assessing risk of bias in RCT trials (13). This tool evaluates the presence of random sequence generation, allocation concealment, blinding of participants and personnel, blinding of outcome assessment, incomplete outcome data, selective reporting, and other risks of confounding. Publication bias was assessed using Begg's adjusted rank correlation test.

### Statistical analysis

Meta-analysis was performed using Review Manager software (version 5.4.1) and STATA 12.0 software. Continuous data were analyzed using mean differences or standardized mean differences with 95% confidence intervals (CIs). Recalculation of the pooled effect estimates using the original metric used by the meta-analysis study authors [hazard ratio (HR), and risk ratio (RR) with 95% CI]. Heterogeneity was assessed using the *I^2^* test. A random-effects model was applied if significant heterogeneity was detected (*I*^2^ > 50%). When heterogeneity is high, we conduct subgroup analyses or sensitivity analyses. Otherwise, a fixed-effects model was utilized. Weighted mean differences (WMD) with 95% CIs were used to evaluate outcomes. Funnel plot analysis was performed to assess publication bias. Statistical significance was set at *P* < 0.05.

## Results

### Study selection

**Figure 1:**
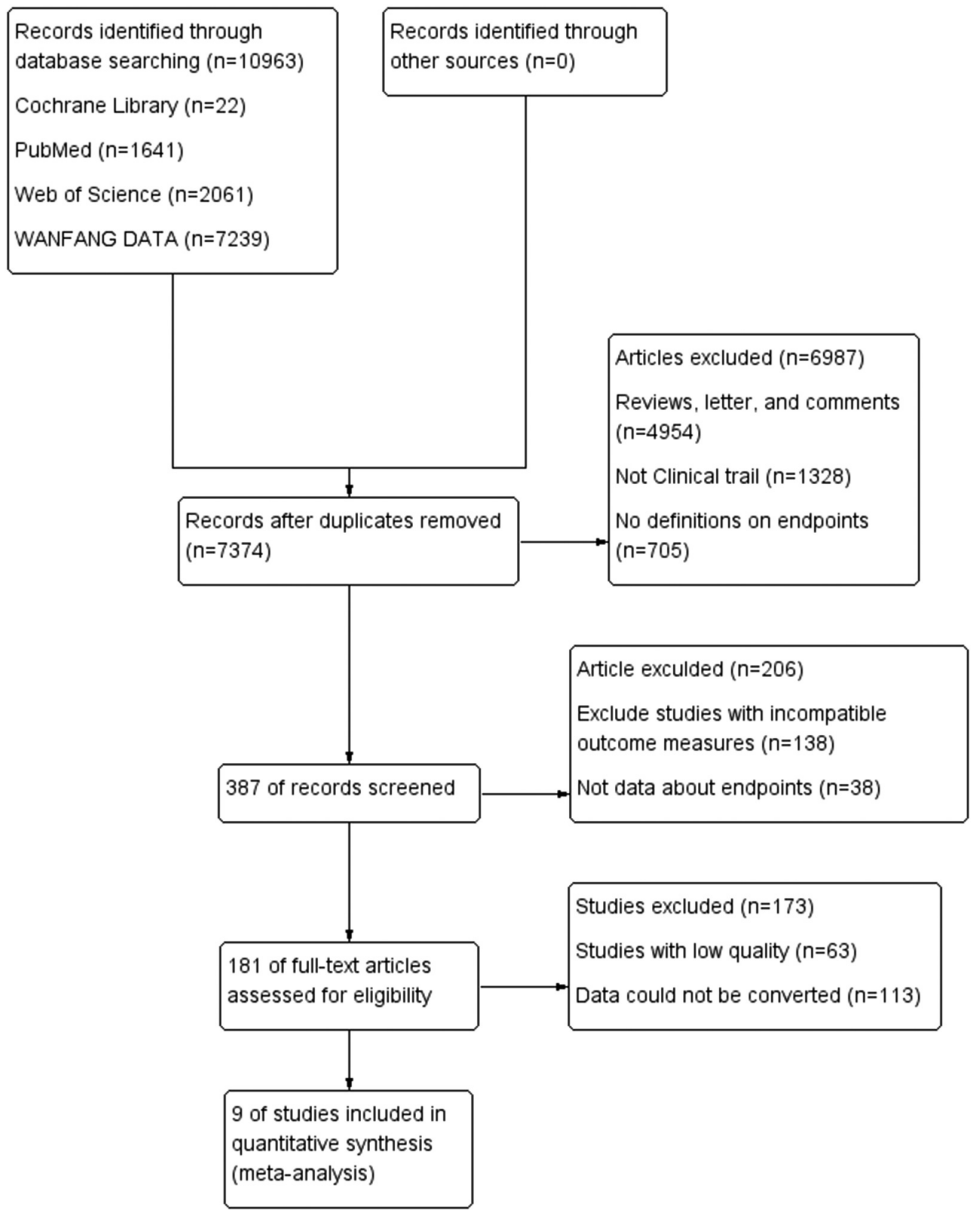
Study flow diagram.

Per the flowchart, the system search generated 10,963 records from databases (Cochrane Library, PubMed, Web of Science, and WAMFANG). After removing duplicates, 7,374 records remained. Following exclusions of 4,954 records including reviews, letters, and comments, 1,328 non-process trials, and 705 records lacking endpoint definitions, 387 records were selected for full-text review. After excluding studies with incompatible outcome metrics, low-quality studies, and data conversion issues, 9 studies were ultimately included in the meta-analysis (Figure 1).

This study included a total of nine RCTs: RALES (The Effect of Spironolactone on Morbidity and Mortality in Patients with Severe Heart Failure) (1), which evaluated spironolactone; EPHESUS (Eplerenone, a Selective Aldosterone Blocker, in Patients with Left Ventricular Dysfunction after Myocardial Infarction) (14), which assessed eplerenone; EMPHASIS-HF (Eplerenone in Patients with Systolic Heart Failure and Mild Symptoms) (15), also focusing on eplerenone; Aldo-DHF (Effect of Spironolactone on Diastolic Function and Exercise Capacity in Patients With Heart Failure With Preserved Ejection Fraction) (16), which examined spironolactone; TOPCAT (Spironolactone for Heart Failure with Preserved Ejection Fraction) (17), again evaluating spironolactone; FIGARO-DKD (Finerenone in Reducing Cardiovascular Mortality and Morbidity in Diabetic Kidney Disease) (18) and FIDELIO-DKD (Finerenone in Reducing Kidney Failure and Disease Progression in Diabetic Kidney Disease) (19), FINEARTS-HF (Finerenone in Heart Failure with Mildly Reduced or Preserved Ejection Fraction) (3), and ARTS (The minerAlocorticoid Receptor Antagonist Tolerability Study) (20), all assessing finerenone. These trials collectively involved 33,128 participants, comprising 67.4% males and 32.6% females (Table 1) (Supplementary Table S2).

**Table 1:** Baseline characteristics of patients in each MRAs trials.

Study Year	Number (%)^a^ Age (years)	Included patients	Follow-up	MRAs	Outcome
RALES 1999	1,663 (27) 65 ± 11	HFrEF, LVEF ≤ 35%, K ≤ 5 mmol/L	12 months	Spironolactone 25 mg/days for 8 weeks → 50 mg/days	CV composite events (CV death or HHF or aborted cardiac arrest), CV death, HHF
EPHESUS 2003	6,642 (29) 64 ± 11	HFpEF, LVEF ≤ 40%, K ≤ 5 mmol/L	16 months	Eplerenone 25 mg/days for 4 weeks → 50 mg/days	CV composite events (CV death or HHF), CV death, HHF
EMPHASIS-HF 2011	2,737 (22) 68.7 ± 7.7	HFpEF, LVEF < 35%, K ≤ 5 mmol/L	21 months	Eplerenone 25 mg/days for 4 weeks → 50 mg/days	CV composite events (CV death or FHHF), CV death, HHF
Aldo-DHF 2013	422 (52) 67 ± 8	HFpEF, LVEF ≥ 50%, K ≤ 5 mmol/L	11.6 months	Spironolactone 25 mg/days	Cardiac hospitalization, Noncardiac hospitalization, Worsening coronary heart disease, New or worsening anemia
TOPCAT 2014	3,445 (52) 68 ± 9	HFpEF, LVEF ≥ 45%, K ≤ 5 mmol/L	3.3 years	Spironolactone 15 mg/days → 45 mg/days during 4 months	CV composite events (CV death or HHF), CV death, HHF
FIGARO-DKD 2022	7,352 (31) 65 ± 9	T2DM, CKD, LVEF ≥ 40%, K ≤ 5 mmol/L	3.4 years	Finerenone 10 or 20 mg/days	CV composite events (CV death or HHF), CV death, HHF
FIDELIO-DKD 2020	5,674 (30) 65 ± 9	T2DM, CKD, LVEF ≥ 40%, K ≤ 5 mmol/L	2.6 years	Finerenone 10 or 20 mg/days	CV composite events (CV death or HHF), CV death, HHF
FINEARTS-HF2024	6,001 (46) 72 ± 9	HFpEF, HFmrEF, LVEF ≥ 40% K ≤ 5 mmol/L	32 months	Finerenone 20 or 40 mg/days	CV composite events (CV death or Total worsening heart failure events), CV death, HHF
ARTS-HF2013	458 (21) 71 ± 8	HFrEF, CKD, LVEF ≤ 40%, K ≤ 5 mmol/L	4 weeks	Finerenone 2.5, 5 or 10 mg/days	Change in serum potassium concentration, safety, tolerability, and renal effects

CV, cardiovascular; HF: heart failure; HFpEF, heart failure with preserved ejection fraction; HFrEF, heart failure with reduced ejection fraction; HHF, heart failure hospitalization; FHHF, first hospitalization for heart failure; K, potassium; LVEF, left ventricular ejection fraction; MRAs, mineralocorticoid receptor antagonists; T2DM, type 2 diabetes mellitus.

^a^ Indicates proportion of female.

### Outcomes

#### The impact of MRAs in patients with HFpEF and HFmrEF

**Figure 2:**
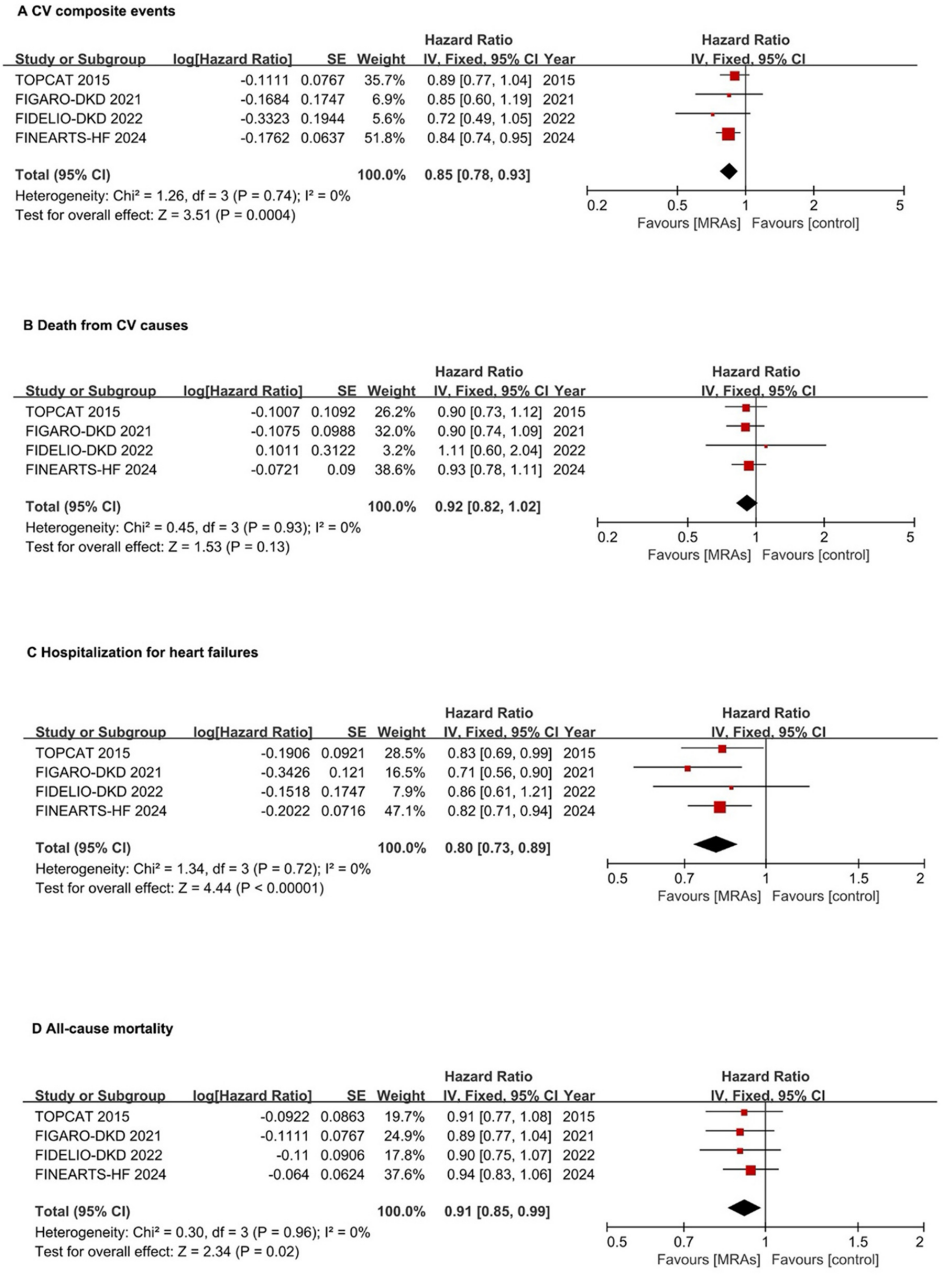
Forest plots assessing MRA effectiveness in hFpEF and hFmrEF patients. **(A)** CV composite events; **(B)** CV death; **(C)** Hospitalization for heart failures; **(D)** All-cause mortality. CI, confidence interval; CV, cardiovascular; HFpEF, heart failure with preserved ejection fraction; HFmrEF, heart failure with mildly reduced ejection fraction; IV, inverse variance; MRA, mineralocorticoid receptor antagonists.

The research analyzed four studies with 7,008 patients to assess the composite primary endpoints (3, 17–19). In the analysis of CV composite events, which encompassed the time to first hospitalization for HF or CV death, MRAs were associated with a significant reduction in risk compared with placebo in patients with HFpEF and HFmrEF. The pooled HR of 0.85 (95% CI: 0.78–0.93; *P* = 0.0004) indicated a 15% relative risk reduction without heterogeneity (*I*^2^ = 0%), suggesting consistent treatment effects across studies (Figure 2A). MRAs showed no significant impact on CV death (pooled HR: 0.92, 95% CI: 0.82–1.02, *P* = 0.13) with low heterogeneity (*I*^2^ = 0%) (Figure 2B). MRAs reduced the risk of hospitalization for HF by 20% (pooled HR: 0.80, 95% CI: 0.73–0.89, *P* < 0.00001) without heterogeneity (*I*^2^ = 0%) (Figure 2C). For all-cause mortality, the pooled analysis demonstrated a significant reduction with MRAs, with an HR of 0.91 (95% CI: 0.85–0.99; *P* = 0.02), indicating a 9% relative risk reduction without heterogeneity (Figure 2D).

### Subgroup analysis

**Figure 3:**
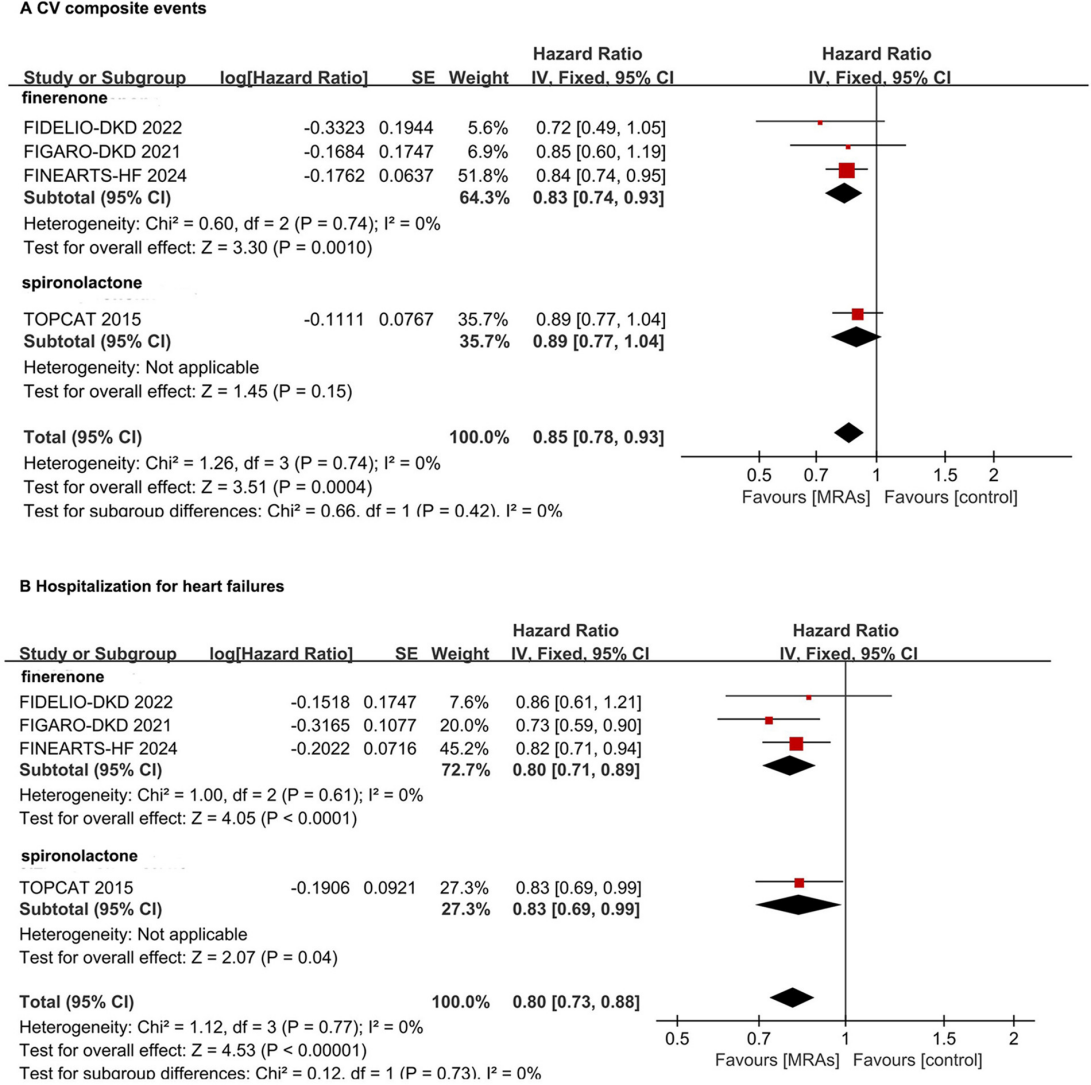
Subgroup analysis of non-steroidal MRA and steroidal MRA in hFpEF and hFmrEF patients. **(A)** CV composite events; **(B)** Hospitalization for heart failures. CI, confidence interval; CV, cardiovascular; HFpEF, heart failure with preserved ejection fraction; HFmrEF, heart failure with mildly reduced ejection fraction; IV, inverse variance; MRA, mineralocorticoid receptor antagonists.

In the CV composite events, the non-steroidal MRA drug finerenone significantly reduced CV composite events by 17% (HR: 0.83, 95% CI: 0.74–0.93, *P* = 0.001). The steroidal MRA drug spironolactone showed no statistically significant reduction in CV composite events for HFmrEF/HFpEF patients (HR: 0.89, 95% CI: 0.77–1.04, *P* = 0.15) (Figure 3A). For the risk of the hospitalization for HF, the non-steroidal MRA drug finerenone significantly reduced HF hospitalizaiton events by 20% (HR: 0.80, 95% CI: 0.71–0.89, *P* < 0.0001). The steroidal MRA drug spironolactone showed statistically significant reduction in HF hospitalizaiton events (HR: 0.83, 95% CI: 0.69–0.99, *P* = 0.04) (Figure 3B).

### The impact of MRAs in patients with HFrEF

**Figure 4:**
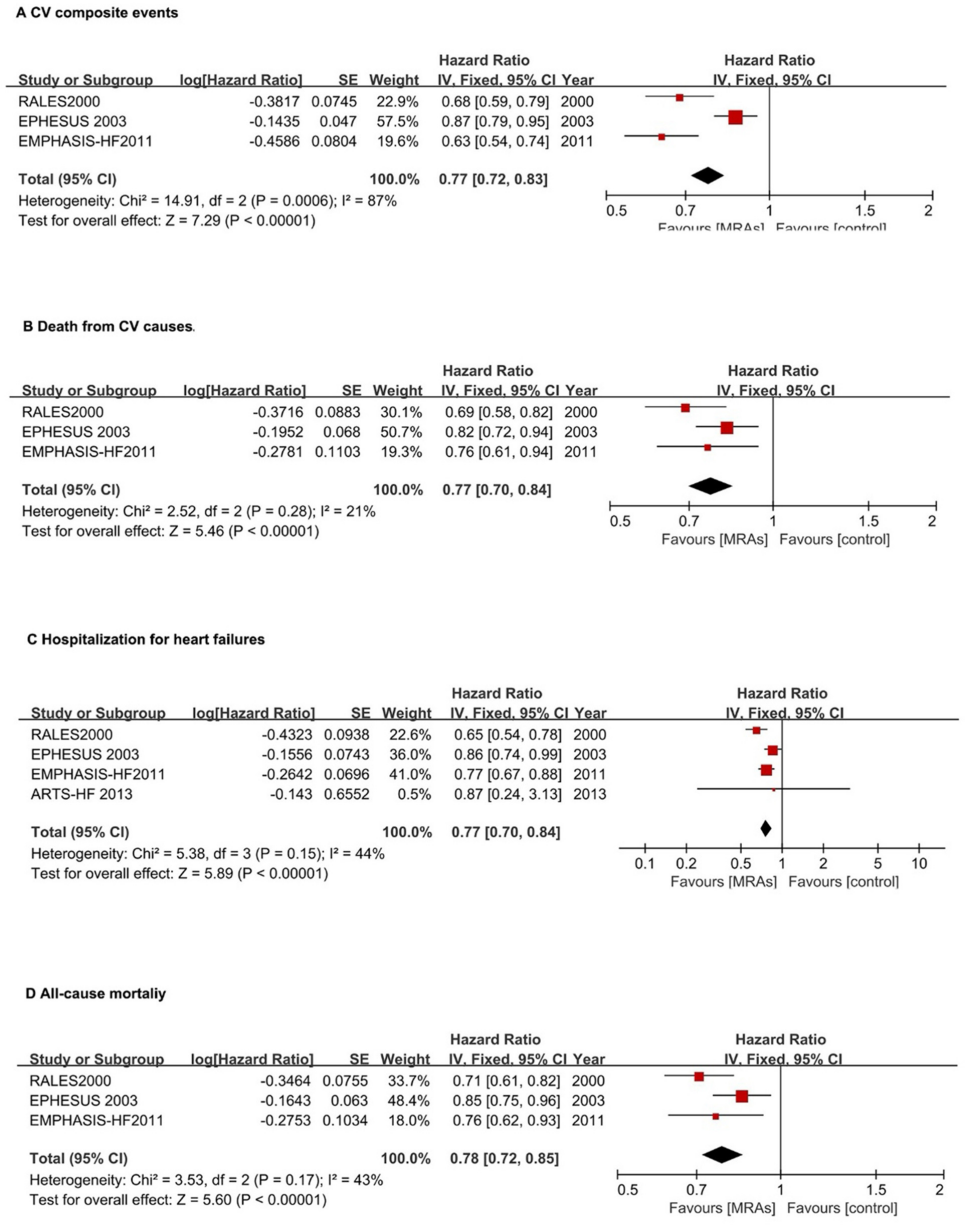
Forest plots assessing MRA effectiveness in hFrEF patients.**(A)** CV composite events; **(B)** CV death; **(C)** Hospitalization for heart failures; **(D)** All-cause mortality. CI, confidence interval; CV, cardiovascular; HFpEF, heart failure with preserved ejection fraction; HFrEF, heart failure with reduced ejection fraction; IV, inverse variance; MRA, mineralocorticoid receptor antagonists.

In patients with HFrEF, MRAs reduced the risk of CV composite events (first HF hospitalization or CV mortality) by 23% (pooled HR: 0.77, 95% CI: 0.72–0.83, *P* < 0.00001). However, high heterogeneity was observed (*I*² = 87%, *P* = 0.0006) (Figure 4A).

For CV mortality, MRAs reduced risk by 23% (pooled HR: 0.77, 95% CI: 0.70–0.84, *P* < 0.0001) with low heterogeneity (*I*² = 21%) (Figure 4B).

MRAs significantly reduced HF hospitalization risk by 23% (pooled HR: 0.77, 95% CI: 0.70–0.84, *P* < 0.00001) with moderate heterogeneity (*I*² = 44%) (Figure 4C). For all-cause mortality, MRAs reduced risk by 22% (HR: 0.78, 95% CI: 0.72–0.85; *P* < 0.00001) with moderate heterogeneity (*I*² = 43%) (Figure 4D).

**Figure 5:**
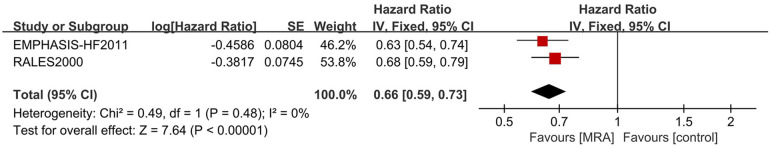
Sensitive analysis of MRA in hFrEF patients. IV, inverse variance; MRA, mineralocorticoid receptor antagonists.

For CV endpoints, a sensitivity analysis was executed. The exclusion of the EPHESUS trial culminated in a marked reduction in heterogeneity (*I*^2^ = 0). This outcome underscores the EPHESUS trial as the principal determinant of the heterogeneity (Figure 5).

### Adverse events associated with MRAs

**Figure 6:**
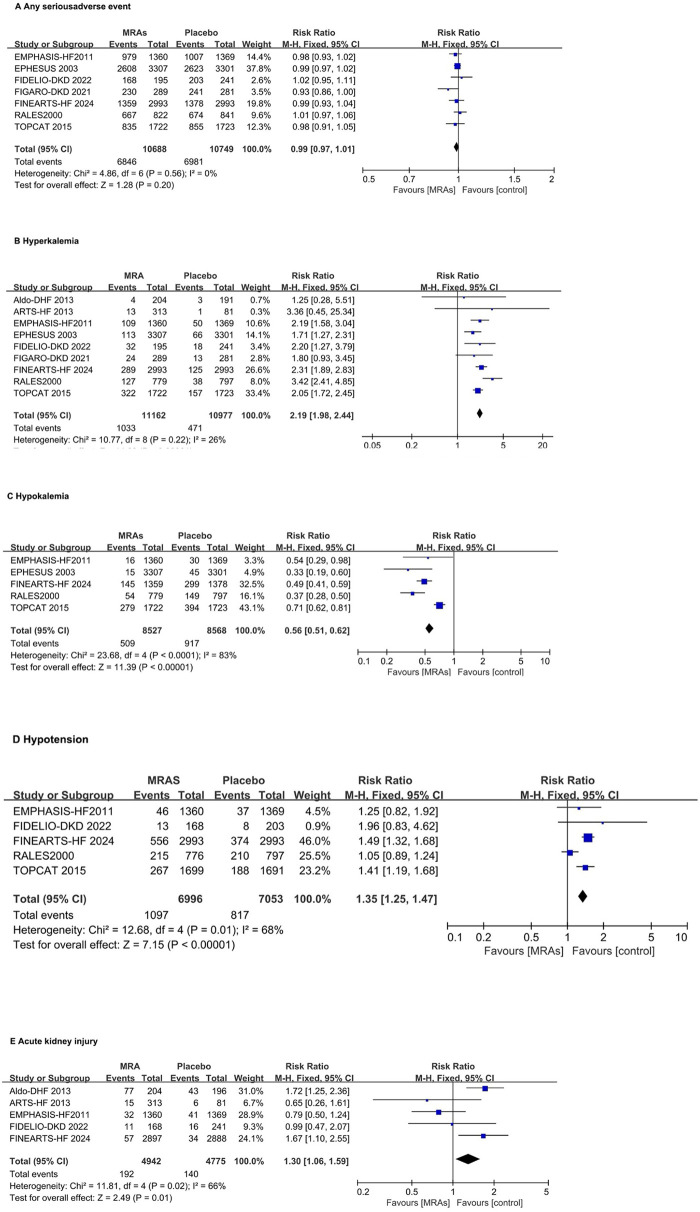
Forest plots assessing MRA safety. **(A)** Any serious adverse event; **(B)** Hyperkalemia; **(C)** Hypokalemia; **(D)** Hypotension; **(E)** Acute kidney injury. MRA, mineralocorticoid receptor antagonists.

The RR for any serious adverse event with MRAs vs. placebo is 0.99 (95% CI: 0.97–1.01; *P* = 0.20) without heterogeneity (Figure 6A). MRAs significantly increase the risk of hyperkalemia, with an RR of 2.19 (95% CI: 1.98–2.44; *P* < 0.00001) with mild heterogeneity (*I*^2^ = 26%) (Figure 6B). The RR for hypokalemia with MRAs is significantly reduced to 0.56 (95% CI: 0.51–0.62; *P* < 0.00001) with high heterogeneity (*I*^2^ = 83%) (Figure 6C). The risk of hypotension is significantly increased with MRAs (RR: 1.35, 95% CI: 1.15–1.47, *P* < 0.00001) with moderate heterogeneity (*I*² = 68%) (Figure 6D). The risk of acute kidney injury is significantly increased with MRAs (RR: 1.30, 95% CI: 1.06–1.59, *P* = 0.01) with moderate heterogeneity (*I*² = 66%) (Figure 6E).

Subgroup analysis of hyperkalemia, MRAs revealed a higher risk of hyperkalemia across all MRA drugs, categorized into finerenone, spironolactone, and eplerenone. Notably, the spironolactone group exhibited significant statistical heterogeneity (*I*^2^ = 72%) (Supplementary Figure S1).

### MRAs' differential impact on HFrEF vs. HFmrEF/HFpEF

**Figure 7:**
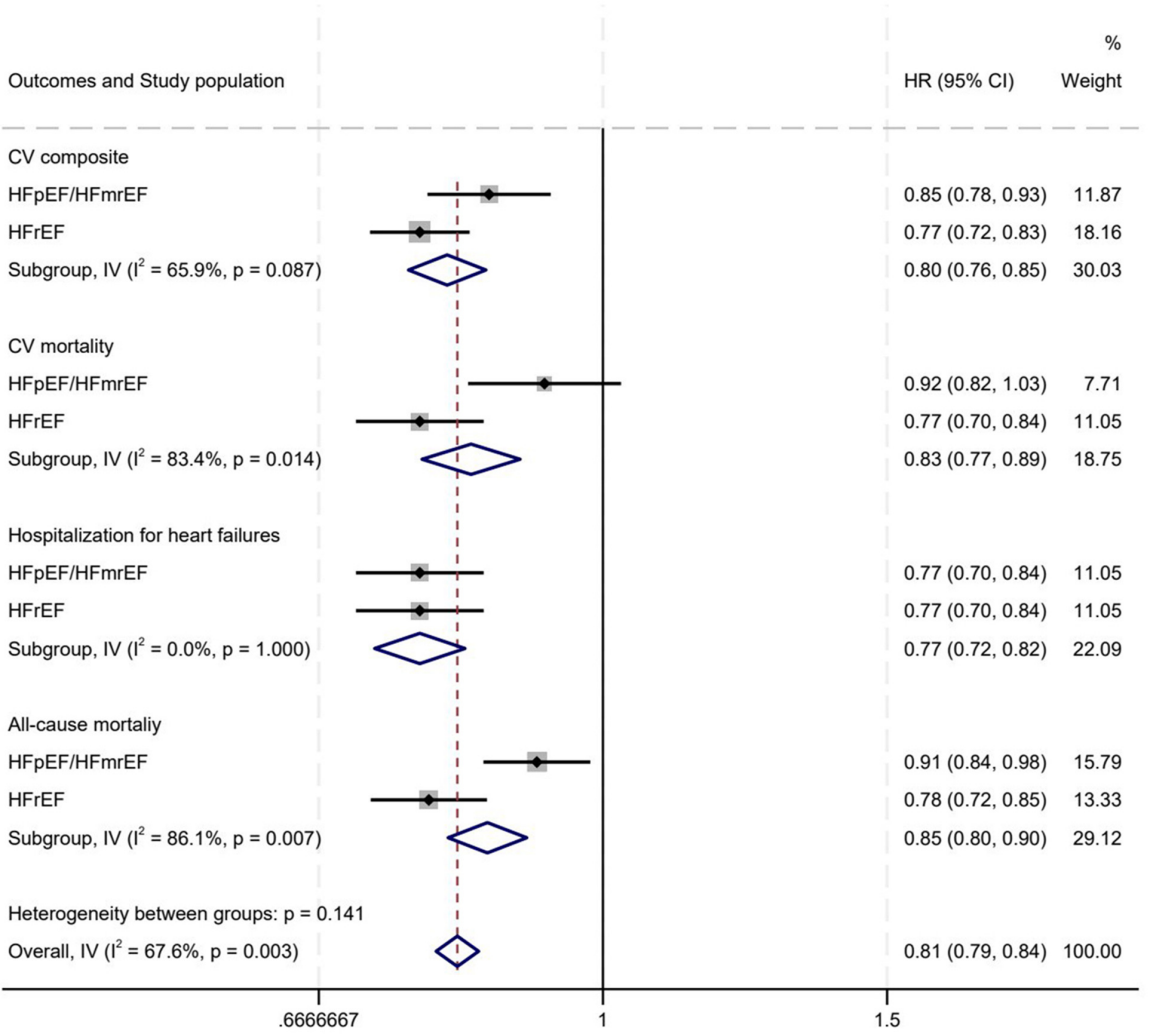
MRAs' differential impact on hFrEF vs. HFmrEF/HFpEF. **(A)** CV composite events; **(B)** CV death; **(C)** Hospitalization for heart failures; **(D)** All-cause mortality. HR, hazard ratio; CV, cardiovascular; HFpEF, heart failure with preserved ejection fraction; HFmrEF, heart failure with mildly reduced ejection fraction; IV, inverse variance; MRA, mineralocorticoid receptor antagonists.

For the CV endpoint, MRAs significantly lowered the HR to 0.85 (95% CI: 0.78–0.93) in the HFpEF/HFmrEF subgroup and to 0.77 (95% CI: 0.72–0.83) in the HFrEF subgroup, exhibiting considerable heterogeneity (*I*^2^ = 65.9%, *P* = 0.007). In terms of CV mortality, the HR for MRA was 0.92 (95% CI: 0.82–1.03) in the HFpEF/HFmrEF subgroup and 0.77 (95% CI: 0.70–0.84) in the HFrEF subgroup, with significant heterogeneity (*I*^2^ = 83.4%, *P* = 0.014). For heart failure hospitalization, the HR with MRA was 0.80 (95% CI: 0.72–0.88) in the HFpEF/HFmrEF subgroup and 0.77 (95% CI: 0.70–0.84) in the HFrEF subgroup, with no significant heterogeneity (*I*^2^ = 0%, *P* = 0.578). Regarding all-cause mortality, the HR for MRA was 0.91 (95% CI: 0.84–0.98) in the HFpEF/HFmrEF subgroup and 0.78 (95% CI: 0.72–0.85) in the HFrEF subgroup, showing significant heterogeneity (*I*^2^ = 86.1%, *P* = 0.007) (Figure 7).

### Risk of bias and publication bias

The risk of bias assessment suggests that most trials included in the meta-analysis are at a low risk of bias across most domains, which enhances the credibility of the findings. However, the high risk of bias in TOPCAT study, particularly in allocation concealment and other bias, may affect the interpretation of its results and should be considered when assessing the overall evidence (Supplementary Figure S2). The forest plot analysis revealed a low likelihood of publication bias (Supplementary Figure S3–S5).

## Discussion

Our analysis of MRA treatment in HFrEF patients corroborates findings from major studies such as RALES and EMPHASIS-HF, highlighting its substantial benefits in decreasing hospitalization rates and mortality across all EF spectum, with notable effects in HFrEF cases. This reinforces MRA's pivotal role as a therapeutic strategy in managing HF.

The mechanisms behind the varying efficacy of MRAs in HFpEF and HFrEF are multifaceted, involving both cardiac and extracardiac factors (21). HFpEF and HFrEF exhibit distinct pathophysiological mechanisms, which in turn influence the effectiveness of MRAs in these conditions (22, 23). While MRAs have shown clear efficacy in HFrEF, their role in HFpEF remains well-defined, largely clear due to the complex and heterogeneous nature of HFpEF (24). MRAs block the MR, curbing myocardial fibrosis and fluid retention. In HFrEF, characterized by heightened neurohormonal activity like renin-angiotensin-aldosterone system (RAAS) and sympathetic overdrive, MRAs are notably effective due to their impact on contractility and remodeling (25). HFpEF, often tied to conditions like obesity and diabetes, involves adipose tissue releasing inflammatory cytokines. This leads to coronary microvascular inflammation and diminished nitric oxide levels, causing myocardial stiffness and diastolic dysfunction. The complexity of HFpEF's pathophysiology means that MR blockade alone is insufficient to halt its progression effectively (26).

In HFrEF, ventricular function in HFrEF is characterized by impaired contraction, whereas in HFpEF, it is marked by impaired relaxation. This distinction is critical as it influences the efficacy of MRAs, which primarily target fluid retention and blood pressure regulation—mechanisms that are more relevant to systolic dysfunction observed in HFrEF (27). Furthermore, calcium handling differs between two conditions: HFrEF is associated with disrupted t-tubule structure and reduced Ca²^+^release, leading to impaired systolic function, whereas HFpEF maintains t-tubule structure but exhibits diastolic Ca²^+^ irregularities, resulting in increased myocardial stiffness. These differences may clarify why MRAs tend to be more effective in HFrEF (27). Additionally, systemic inflammation and comorbidities play a significant role in HFpEF, which is often associated with hypertension, diabetes, and obesity, which contribute to coronary microvascular dysfunction and myocardial hypertrophy—factors that are not directly targeted by MRAs. Such associations may explain the limited efficacy of MRAs in HFpEF compared to HFrEF (28). Vascular and metabolic factors significantly affect HFpEF by altering ventricular-vascular coupling and increasing arterial stiffness, primarily due to aging and comorbidities. Since these extracardiac factors are not the main targets of MRAs, the effectiveness of MRAs in treating HFpEF is further limited (29).

MRAs, including spironolactone and eplerenone, play a crucial role in managing CV diseases such as HF and hypertension. Despite therapeutic benefits, they are associated with side effects such as hyperkalemia, renal dysfunction, and hormonal disturbances, particularly with spironolactone. Addressing these adverse effects is vital for optimizing MRA therapy outcomes.

Hyperkalemia, a significant side effect of MRAs, poses a particular risk for patients with CKD or those concurrently using RAAS inhibitors (30). Subgroup analysis revealed the heterogeneity in hyperkalemia risk among spironolactone users correlates with study-specific dosages and patient demographics. As a first-generation, non-selective MRA, spironolactone leads to metabolic issues with varying impacts across age groups. Its main metabolite, canrenone, is a potent MR antagonist with a long half-life of 10–35 h, increasing the risk of hyperkalemia due to prolonged potassium retention. In the RALE study (1), targeting elderly patients (79 years old) with severe heart failure (NYHA Class IV), the highest spironolactone dosage (25–50 mg/day) was used, raising hyperkalemia risk. The TOPCAT study (17), focusing on HFpEF and hypertensive patients, used a moderate dosage (15–45 mg/day). The Aldo-DHF study (16), with a stable dosage (25 mg/day), involved patients with isolated HFpEF and preserved renal function.

Our analysis supports a tiered monitoring approach for hyperkalemia in HF patients, with a focus on initial screening and ongoing surveillance. Key groups for monitoring include those over 65, with estimated glomerular filtration rate (eGFR) < 45 ml/min, CKD, diabetes, or prior hyperkalemia. Initiate MRA therapy only if serum potassium is ≤5.0 mmol/L, with subsequent checks at 1–2 weeks, monthly, and annual eGFR and electrolyte assessments for CKD patients (31, 32). Adjust therapy based on potassium levels: continue MRA at 5.1–5.5 mmol/L with potassium supplement or NSAID discontinuation, potassium-restricted diet, and new binders; reduce MRA by 50% at 5.6–6.0 mmol/L with binders; cease MRA immediately if potassium exceeds 6.0 mmol/L, initiate urgent potassium-lowering measures, and restart at a lower dose once levels return to ≤5.0 mmol/L (33). Additionally, co-treatment with sodium-glucose cotransporter 2 (SGLT2) inhibitors may mitigate hyperkalemia risk due to their diuretic properties (30).

MRAs can trigger acute kidney function decline, notably in individuals with existing renal issues. It is essential to evaluate renal function before beginning MRA treatment and to perform ongoing monitoring. If significant impairment is detected, dosage adjustment or drug withdrawal may be warranted (34). Spironolactone, a steroidal MRA, may induce sex-related side effects including gynecomastia in men and menstrual irregularities in women, attributed to its anti-androgenic and progesterone-like activities (34). Switching to eplerenone, a more selective MRA with fewer hormonal side effects, can help mitigate these issues (34). Among patients with CKD, MRAs have demonstrated advantages in decreasing CV mortality and proteinuria; however, their application is frequently constrained due to the hyperkalemia risk (35). Developing non-steroidal MRAs holds promise for diminished side effects while preserving therapeutic efficacy, potentially broadening the clinical application of MRAs (34). Non-steroidal MRAs, distinct from steroidal MRAs that are progesterone derivatives, feature a unique chemical structure. This distinction endows non-steroidal MRAs with greater selectivity and affinity for the MR, minimizing off-target effects and enhancing safety (36). Furthermore, steroidal MRAs can lead to side effects like gynecomastia due to their impact on multiple nuclear receptors. Non-steroidal MRAs, with their higher selectivity, are less likely to cause such hormonal issues (37).

Finerenone, a non-steroidal MRA, exhibits a balanced effect on both the heart and kidneys, unlike spironolactone, which is more kidney-focused (38). This difference is complemented by finerenone's unique pharmacokinetic characteristics, including a shorter half-life and distinct metabolic pathways, which enhance its safety profile (39). Additionally, finerenone demonstrates significant anti-inflammatory and anti-fibrotic properties that are essential for cardiorenal health, with particular efficacy in mitigating renal inflammation and fibrosis more effectively than eplerenone (36). Compared to steroidal MRAs, non-steroidal MRAs like finerenone pose a lower risk of hyperkalemia, rendering them a safer option for patients with CKD and those concurrently using RAAS inhibitors (37). This advantage is further underscored by the effectiveness of non-steroidal MRAs in reducing cardiovascular and renal event rates among patients with CKD and type 2 diabetes. Notably, clinical trials have shown that finerenone significantly slows the progression of kidney disease and decreases cardiovascular events (39).

Furthermore, the potential of non-steroidal MRAs to provide synergistic benefits when used in combination with other therapies, such as SGLT2 inhibitors, is currently under investigation. Such combinations may further reduce the risk of hyperkalemia and strengthen cardiorenal protection, offering promising avenues for future treatment strategies (37, 40).

Non-steroidal MRAs present several advantages over steroidal MRAs, including enhanced safety and efficacy profiles. However, these agents are not without limitations. Although the risk of hyperkalemia is diminished, it remains a concern necessitating vigilant monitoring. Furthermore, the long-term effects and potential benefits of non-steroidal MRAs across a broader range of patient populations remain under investigation. As ongoing research unfolds, these agents may emerge as a pivotal component in the management of cardiorenal diseases, particularly for patients at elevated risk of adverse outcomes with conventional therapies.

### Study strengths and limitations

This study marks the first comprehensive meta-analysis to evaluate the efficacy of MRAs in HF patients, spanning across HFrEF, HFmrEF, and HFpEF categories. Beyond the comparative analysis of MRAs within EF subgroups, we have also performed a direct “head-to-head” assessment of both the efficacy and safety profiles of steroidal vs. nonsteroidal MRAs concerning specific critical endpoints.

Several of our findings demonstrate notable heterogeneity. To ensure consistency, we conducted sensitivity and subgroup analyses on those results exhibiting substantial heterogeneity in order to identify its root causes. Among patients with HFrEF, the primary cardiovascular composite endpoint revealed significant heterogeneity. A sensitivity analysis that excluded the EPHESUS trial indicated no heterogeneity, thereby highlighting EPHESUS as a principal contributor. The EPHESUS trial's composite endpoint encompassed cardiovascular mortality or the first hospitalization for cardiovascular events, contrasting with EMPHASIS-HF, which included cardiovascular mortality or the first hospitalization for heart failure. The employment of spironolactone in the RALES trial suggests that differences in medication may also play a role.

## Data Availability

The raw data supporting the conclusions of this article will be made available by the authors, without undue reservation.

## Supplementary material

Supplementary Figure 1 Subgroup analysis of hyperkalemia. MRA, mineralocorticoid receptor antagonists.

Supplementary Figure 2 Risk of bias analysis for randomized control studies

Supplementary Figure 3 Publication bias of HFpEF and HFmrEF study. ((A) CV composite events; (B) CV death; (C) Hospitalization for heart failures; (D) All-cause mortality. HFpEF, heart failure with preserved ejection fraction; HFmrEF: heart failure with mildly reduced ejection fraction.

Supplementary Figure 4 Publication bias of HFpEF and HFmrEF study. ((A) CV composite events; (B) CV death; (C) Hospitalization for heart failures; (D) All-cause mortality. HFrEF: heart failure with reduced ejection fraction.

Supplementary Figure 5 Publication bias of MRA safety. MRA, mineralocorticoid receptor antagonists. (A) Any serious adverse event; (B) Hyperkalemia; (C) Hypokalemia; (D) Hypotension; (E) Acute kidney injury.
