# Supplementary figures and images for: Mineralocorticoid receptor antagonists in heart failure: a systematic review and meta-analysis
Source: Front Cardiovasc Med. 2025 Sep 1;12:1667236. doi: 10.3389/fcvm.2025.1667236 (PMC12434096; doi:10.3389/fcvm.2025.1667236)

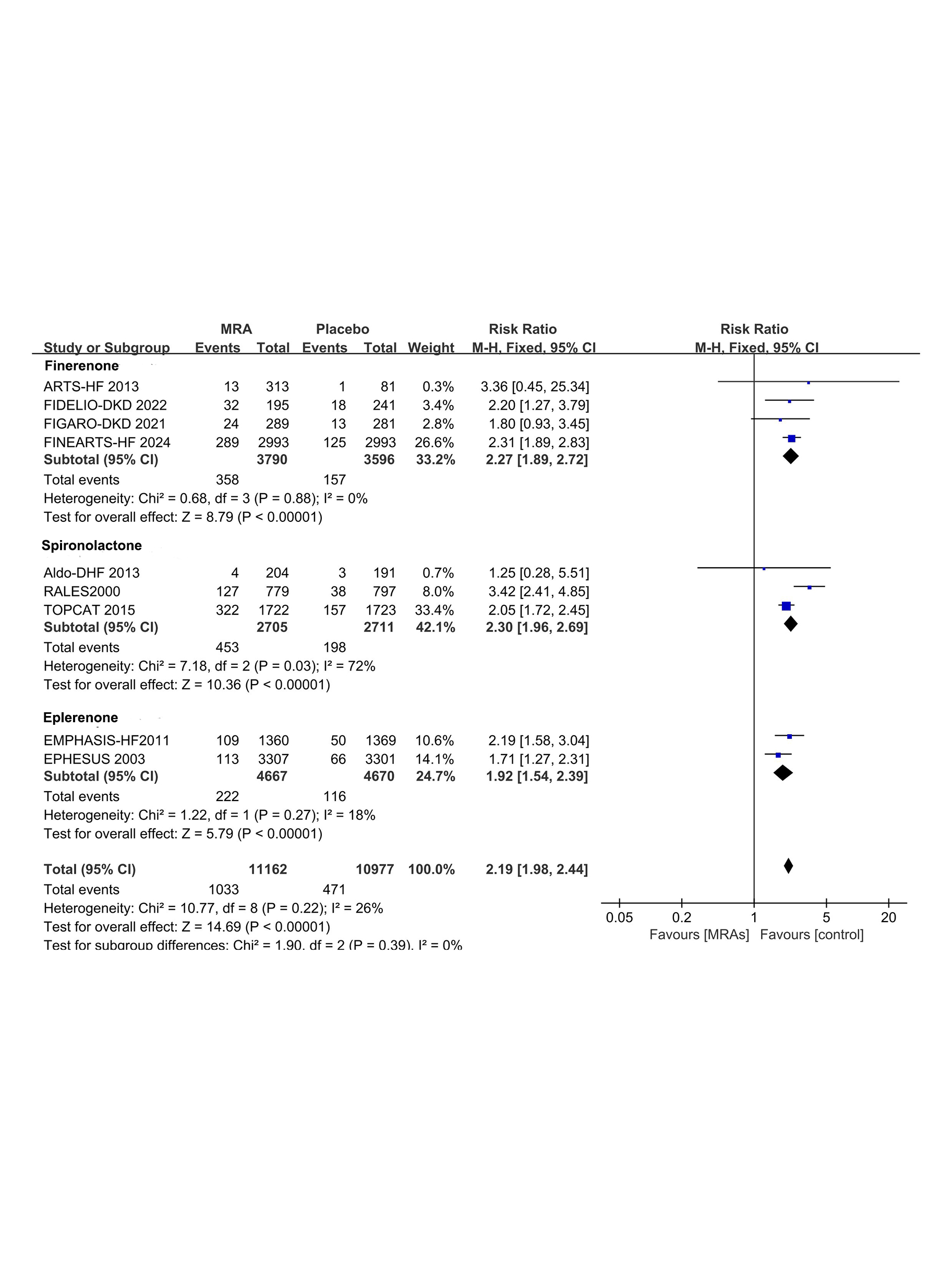

Supplement: Supplementary Figure 1 — Subgroup analysis of hyperkalemia. MRA, mineralocorticoid receptor antagonists. [file Image1.jpeg]

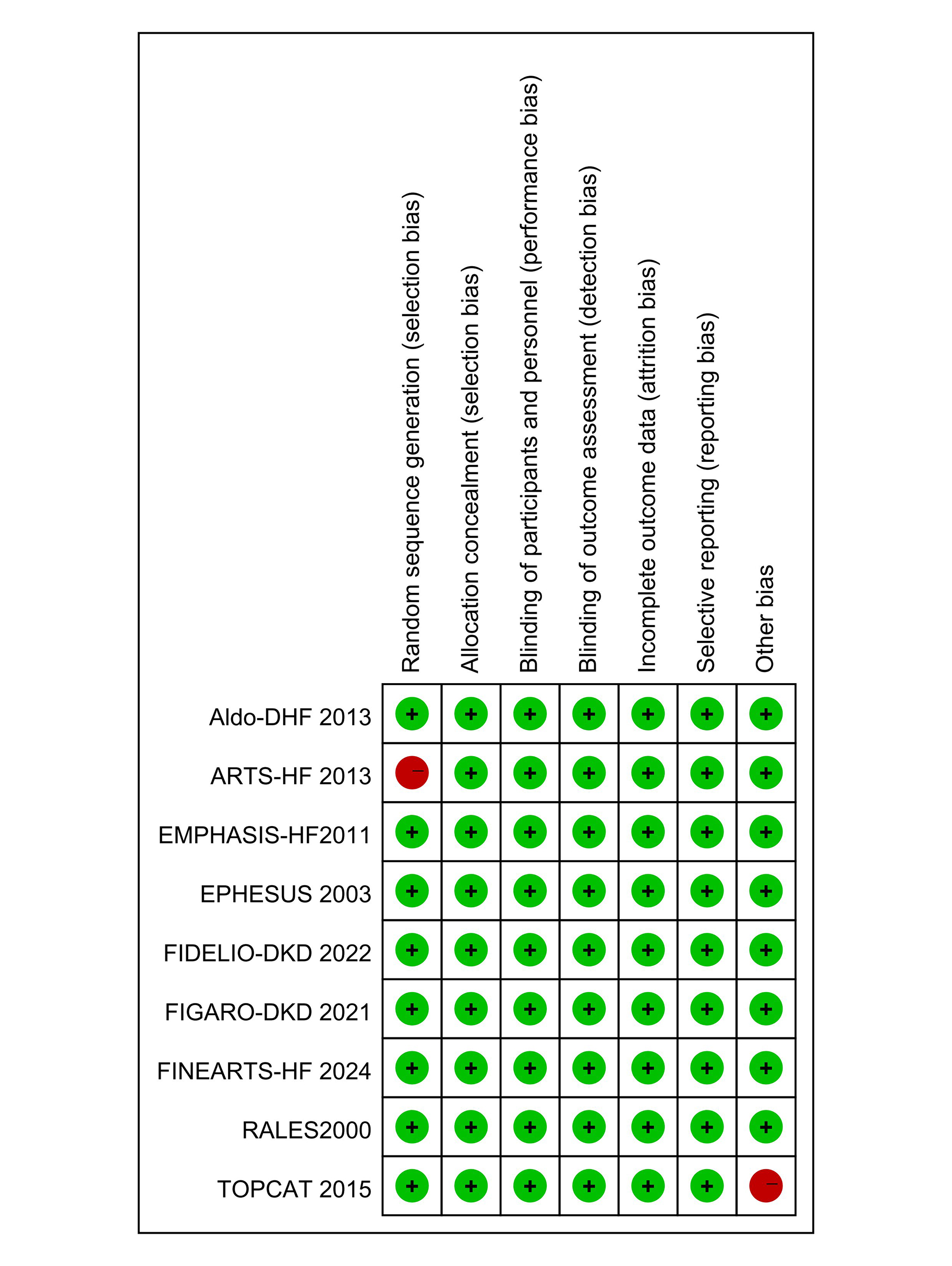

Supplement: Supplementary Figure 2 — Risk of bias analysis for randomized control studies [file Image2.jpeg]

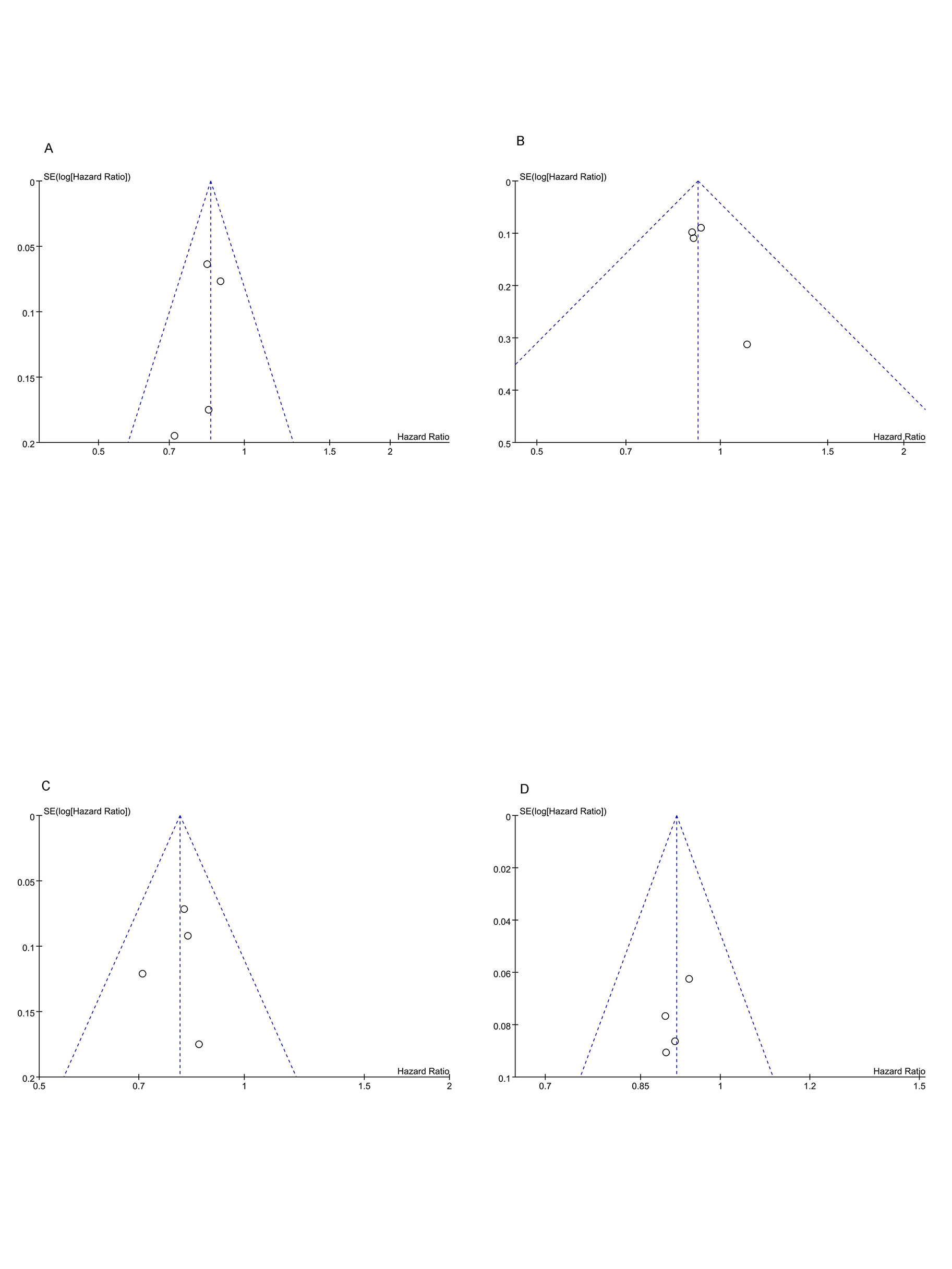

Supplement: Supplementary Figure 3 — Publication bias of HFpEF and HFmrEF study. ((A) CV composite events; (B) CV death; (C) Hospitalization for heart failures; (D) All-cause mortality. HFpEF, heart failure with preserved ejection fraction; HFmrEF: heart failure with mildly reduced ejection fraction. [file Image3.jpeg]

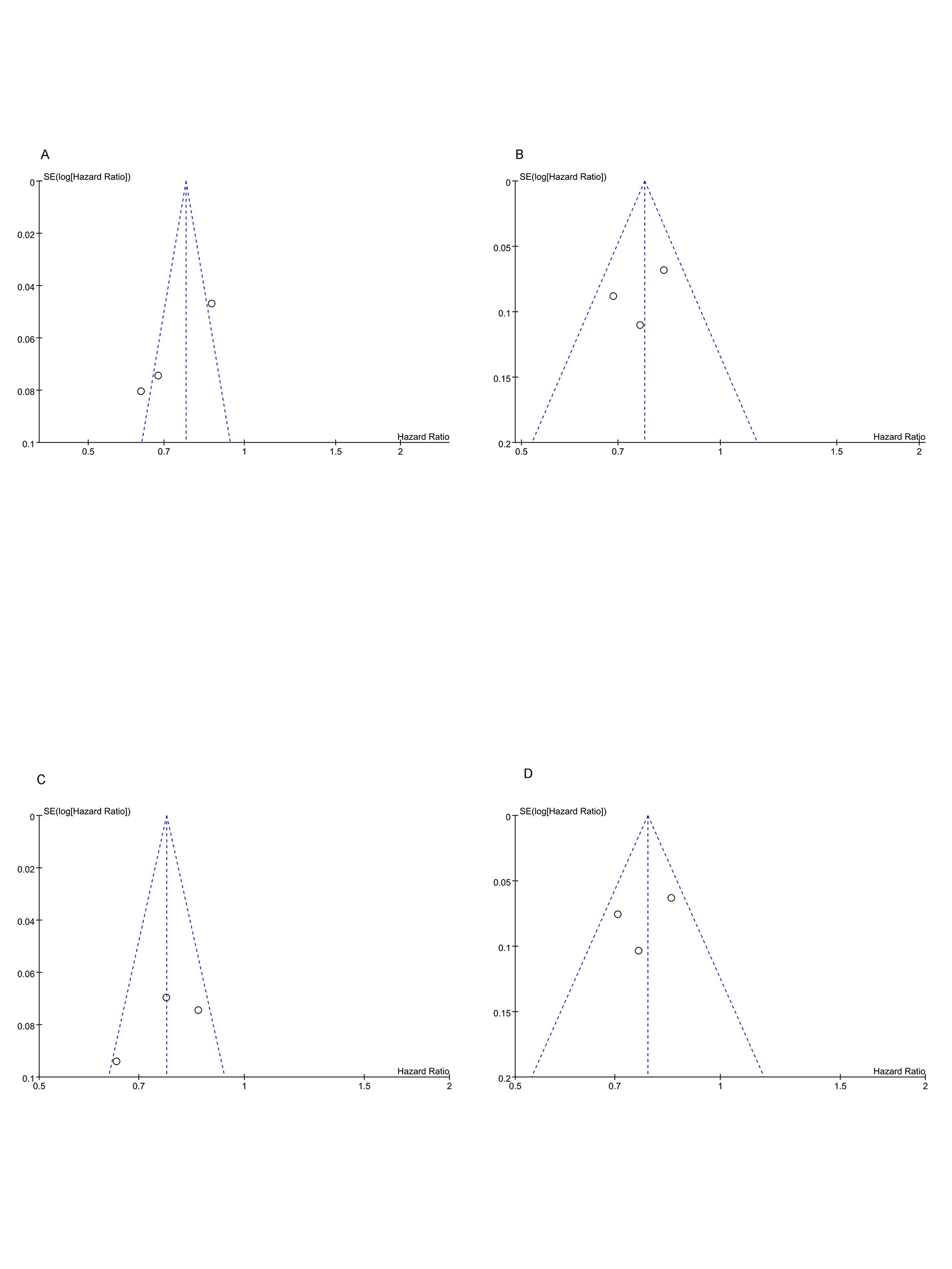

Supplement: Supplementary Figure 4 — Publication bias of HFpEF and HFmrEF study. ((A) CV composite events; (B) CV death; (C) Hospitalization for heart failures; (D) All-cause mortality. HFrEF: heart failure with reduced ejection fraction. [file Image4.jpeg]

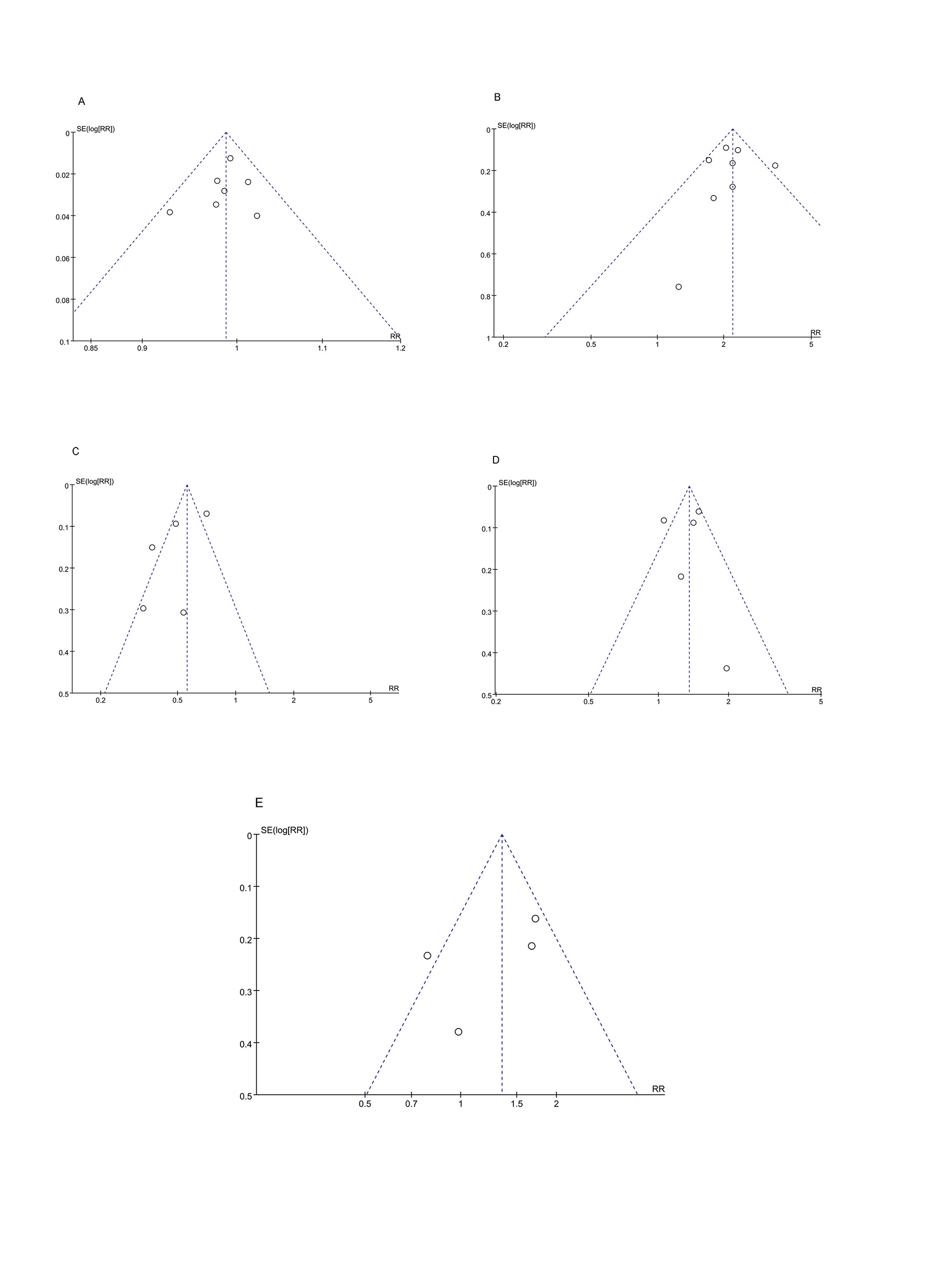

Supplement: Supplementary Figure 5 — Publication bias of MRA safety. MRA, mineralocorticoid receptor antagonists. (A) Any serious adverse event; (B) Hyperkalemia; (C) Hypokalemia; (D) Hypotension; (E) Acute kidney injury. [file Image5.jpeg]
