# Supplementary material for: Mineralocorticoid receptor antagonists in heart failure: a systematic review and meta-analysis
Source: Front Cardiovasc Med. 2025 Sep 1;12:1667236. doi: 10.3389/fcvm.2025.1667236 (PMC12434096; doi:10.3389/fcvm.2025.1667236)
Supplement: Supplementary file 7 [file Table2.docx]

| **Supplementary Table 2 Characteristics of patients** | | | | | | | | | | | | | | | | | | | |
| --- | --- | --- | --- | --- | --- | --- | --- | --- | --- | --- | --- | --- | --- | --- | --- | --- | --- | --- | --- |
| **Characteristics** | **FINEARTS-HF 2024** | | **FIDELIO-DKD 2022** | | **FIGARO-DKD 2021** | | **Aldo-DHF 2013** | | **TOPCAT 2015** | | **Rales 1999** | | **EPHESUS 2003** | | **EMPHSIS-HF2011** | | **ARTS-HF 2015** | | |
|  | Finerenone (n=3003) | Placebo  (n=2998) | Finerenone (n=195) | Placebo  (n=241) | Finerenone (n=290) | Placebo  (n=281) | Spironolactone  (n=213) | Placebo  (n=209) | Spironolactone  (n= 1722) | Placebo  (N=1723) | Spironolactone  (n=822) | Placebo  (n=841) | Eplerenone  (n = 3319) | Placebo  (n=3313) | Eplerenone  (n=1364) | Placebo  (n=1373) | Finerenone (n=264) | Spironolactone (n=63) | Placebo  (n=65) |
| Age, years | 71.9 (9.6) | 72.0 (9.7) | 66.4 (8.8) | | 64.9 (9.0) | 66.3 (8.8) | 67 (8.0) | 67 (8.0) | 68.7 (9.0) | 68.7 | 65.0 (12.0) | 65.0 (12.0) | 64.0 (11) | 64.0 (12) | 68.7 (7.7) | 68.6 (7.6) | 72.1 (40–89) | | |
| Gender^§^ |  |  |  | |  |  |  |  |  |  |  |  |  |  |  |  |  | | |
| Male | 1648 (54.9) | 1621 (54.1) | 120 (61.5) | | 182 (62.8) | 168 (59.8) | 102 (47.9) | 99 (47.0) | 834 (48.4) | 836 (48.5) | 603 (73.0) | 614 (73.0) | 2380 (72.0) | 2334 (70.0) | 1072 (77.6) | 1072 (78.1) | 312 (79.6) | | |
| Female | 1355 (45.1) | 1377 (45.9) | 75 (38.5) | | 108 (37.2) | 113 (40.2) | 111 (52.1) | 110 (53.0) | 888 (51.6) | 887 (51.5) | 219 (27.0) | 227 (27.0) | 939 (28.0) | 979 (30.0) | 309 (22.4) | 301 (21.9) | 80 (20.4) | | |
| Race, n (%) |  |  |  | |  |  |  |  |  |  |  |  |  |  |  |  |  | | |
| White | 2366 (78.8) | 2369 (79.0) | 156 (80.0) | | 263 (90.7) | 239 (85.1) | NA | NA | 1525 (88.6) | 1537 (89.2) | (87)* | (86)* | 2995 (90.0) | 2989 (90.0) | 1127 (82.6) | 1141 (83.1) | NA | | |
| Black/African American | 49 (1.6) | 39 (1.3) | 9 (4.6) | | 15 (5.2) | 11 (3.9) | NA | NA | NA | NA | NA | NA | 30 (1.0) | 44.0 (1.0) | 37 (2.7) | 30 (2.2) | NA | | |
| Asian | 497 (16.6) | 499 (16.6) | 23 (11.8) | | 10 (3.4) | 18 (6.4) | NA | NA | NA | NA | NA | NA | NA | NA | 158 (11.6) | 158 (11.5) | NA | | |
| Others | 91 (3.0) | 91 (3.0) | 7 (3.6) | | 2 (0.7) | 13 (4.6) | NA | NA | NA | NA | NA | NA | 294 (9.0) | 280.0 (8.0) | 42 (3.1) | 44 (3.2) | NA | | |
| SBP, mmHg | 129.5 (15.3) | 129.5 (15.3) | 138.0 (13.6) | | 135.7 (13.6) | 135.0 (13.9) | 135.0 (18.0) | 135.0 (18.0) | 130.0 (9.0) | 130.0 (10.0) | 123.0 (21.0) | 122.0 (20.0) | 119.0 (17.0) | 119.0 (17.0) | 124.0 (17.0) | 124.0 (17.0) | 127.3 (81.0–180.0) | | |
| DBP, mmHg | NA | NA | 76.4 (9.6) | | 77.6 (9.7) | 76.0 (10.5) | 79.0 (10.0) | 80.0 (12.0) | 80.0 (10.0) | 80.0 (10.0) | 75.0 (12.0) | 75.0 (11.0) | 72.0 (11.0) | 72.0 (11.0) | 75.0 (10.0) | 75.0 (10.0) | NA | | |
| LVEF, % | 52.6 (7.8) | 52.5 (7.8) | > 40.0 | | > 40.0 | > 40.0 | > 50.0 | > 50.0 | 56.0 (5.0) | 56.0 (6.0) | 25.6 (6.7) | 25.2 (6.8) | 33.0 (6.0) | 33.0 (6.0) | 26.2 (4.6) | 26.1 (4.7) | ≤ 40.0 | | |
| BMI, kg/m^2^ | 29.9 (6.1) | 30.0 (6.1) | 33.7 (6.6) | | 33.3 (6.4) | 32.4 (5.9) | 28.9 (3.6) | 28.9 (3.6) | 31.0 (4.5) | 31.0 (4.5) | NA | NA | NA | NA | 27.5 (4.9) | 27.5 (4.9) | 28.8 (18.1–46.9) | | |
| NT-proBNP, pg/ml | 1053.0 (467.0, 1937.0) | 1028.0 (433.0,1963.0 ) | NA | | NA | NA | 179.0 (81.0, 276.0) | 148.0 (80.0, 276.0) | 887.0 (537.0, 1634.0) | 1017.0 (627.0, 2258.0) | NA | NA | NA | NA | NA | NA | 1381.5 (22.7–32 349.1) | | |
| eGFR, ml/min/1.73m^2^ | 61.9 (19.4) | 62.3 (20.0) | 42.6 (12.9) | | 64.0 (21.4) | 62.8 (22.0) | 79.0 (19.0) | 78.0 (18.0) | 65.3 (12.7) | 65.5 (12.8) | NA | NA | NA | NA | 71.2 (21.9) | 70.4 (21.7) | 47.0(10.0) | | |
| Serum K^+^, mmol/L | 4.4 (0.5) | 4.4 (0.5) | 4.3 (0.5) | | 4.4 (0.5) | 4.4 (0.5) | 4.2 (0.4) | 4.2 (0.4) | 4.3 (0.3) | 4.3 (0.3) | NA | NA | 4.3 (0.4) | 4.3 (0.5) | NA | NA | NA | | |
| UACR, mg/g | NA | NA | 808.1 (398.3, 1783.3) | | 274.6 (110.7, 745.0) | 246.2 (85.0, 729.2) | NA | NA | NA | NA | NA | NA | NA | NA | NA | NA | NA | | |
| hs-CRP, mg/L | NA | NA | 7.5 (16.0) | | 6.0 (7.9) | 4.8 (8.7) | NA | NA | NA | NA | NA | NA | NA | NA | NA | NA | NA | | |
| CVD | NA | NA | 147 (75.4) | | 197 (67.9) | 195 (69.4) | NA | NA | 1232 (71.5) | 1232 (71.5) | NA | NA | NA | NA | NA | NA | NA | | |
| CAD | 784 (26.1) | 757 (25.3) | 124 (63.6) | | 156 (53.8) | 166 (59.1) | 92 (43.0) | 78 (37.0) | 989 (57.4) | 1034 (60.1) | NA | NA | NA | NA | NA | NA | NA | | |
| AF | 1165 (38.8) | 1128 (37.6) | 48 (24.6) | | 60 (20.7) | 53 (19.0) | 13 (6.0) | 9 (4.0) | 611 (35.5) | 603 (35.1) | NA | NA | NA | NA | 409 (30.0) | 435 (31.7) | 177 (45.2) | | |
| Hypertension | 2640 (87.9) | 2685 (89.6) | 191 (97.9) | | 270 (93.1) | 247 (88.0) | 197 (92.0) | 190 (91.0) | 1567 (91.0) | 1580 (91.9) | NA | NA | 60* | 61* | 910 (66.7) | 909 (66.2) | 261 (66.6) | | |
| Medication use, n (%) | | | | | | | | | | | | | | | | | | | |
| Beta-blocker | 2541 (84.6) | 2554 (85.2) | 142 (72.8) | 188 (78.0) | 204 (70.3) | 202 (72.0) | 146 (69.0) | 156 (75.0) | 1346 (78.2) | 1330 (77.3) | 11* | 10* | 75* | 75* | 1181 (86.6) | 1193 (86.9) | 366 (93.4) | | |
| ACEI | 1083 (36.1) | 1072 (35.8) | 73 (37.4) | 122 (50.6) | 152 (52.4) | 145 (51.6) | 167 (78.0) | 158 (76.0) | 1452 (84.3) | 1448 (84.2) | 95* | 94* | 86* | 87* | 1068 (78.3) | 1055 (76.8) | NA | | |
| ARB | 1047 (34.9) | 257 (8.6) | 123 (63.1) | 120 (49.8) | 137 (47.2) | 136 (48.4) |  |  |  |  | NA | NA | 86* | 87* | 261 (19.1) | 266 (19.4) | NA | | |
| Diuretics | NA | NA | 139 (71.3) | 173 (71.8) | 180 (62.1) | 183 (65.1) | 118 (55.0) | 109 (52.0) | 1401 (81.4) | 1416 (82.3) | NA | NA | 60* | 61* | 1150 (84.3) | 1176 (85.7) | 349 (89.0) | | |
| Loop diuretic | 2618 (87.2) | 2621 (87.4) | 114 (58.5) | 129 (53.5) | 111 (38.3) | 109 (38.8) | NA | NA | NA | NA | 100* | 100* | NA | NA | NA | NA | NA | | |
| Thiazide diuretic | 429 (14.3) | 402 (13.4) | 17 (8.7) | 26 (10.8) | 41 (14.1) | 49 (17.4) | NA | NA | NA | NA | NA | NA | NA | NA | NA | NA | NA | | |
| K^+^ supp | 349 (11.6) | 365 (12.2) | 15 (7.7) | 16 (6.6) | 13 (4.5) | 15 (5.3) | NA | NA | NA | NA | NA | NA | NA | NA | NA | NA | NA | | |
| GLP-1RA | 79 (2.6) | 88 (2.9) | 9 (4.6) | 7 (2.9) | 17 (5.9) | 7 (2.5) | NA | NA | NA | NA | NA | NA | NA | NA | NA | NA | NA | | |
| MACE, n (%) |  |  |  |  |  |  |  |  |  |  |  |  |  |  |  |  |  | | |
| CV mortality | 242 (8.1) | 260 (8.7) | 20 (10.3) | 22 (9.1) | 194 (5.3) | 214(5.8) | NA | NA | 160 (9.3) | 176 (10.2) | 226 (27.5) | 314 (37.3) | 407(12.3) | 483(14.6) | 147(10.8) | 185(13.5) | NA | | |
| HF Hospitalization | 842 (28.0) | 1024 (34.2) | 56 (28.7) | 78 (32.4) | 29 (10.0) | 42 (15.0) | NA | NA | 206 (12.0) | 245 (14.2) | 323(39.3) | 529 (63.0) | 345(10.4) | 391(11.8) | 408(29.9) | 491(35.8) | 13(3.3) | | |
| CV death or HHF (%) | 624 (20.8) | 719 (24.0) | 39 (20.0) | 57 (23.7) | NA | NA | NA | NA | NA | NA | NA | NA | NA | NA | NA | NA | NA | | |
| First HHF (%) | NA | NA | 23 (11.8) | 41 (17.0) | 29 (10.0) | 42 (15.0) | NA | NA | NA | NA | NA | NA | NA | NA | NA | NA | NA | | |
| Hyperkalemia(%) | 289 (9.7) | 125 (4.2) | 32 (16.4) | 18 (7.5) | 24 (8.3) | 13 (4.6) | 4 (2.0) | 3 (1.0) | 322(18.7) | 157(9.1) | 127(16.0) | 38(5.0) | 113 (3.4) | 66 (2.0) | 109 (8.0) | 50 (3.7) | 14(3.6) | | |
| Mean (SD) or median (IQR), or n (percents);SBP, systolic blood pressure;DBP, diastolic blood pressure; LVEF, left ventricular ejection fraction;BMI, body mass index; NT-proBNP, N-terminal pro-brain natriuretic peptide; eGFR, estimated glomerular filtration rate;Serum K^+^, serum potassium;UACR, urinary albumin-to-creatinine ratio;hs-CRP, high-sensitivity C-reactive protein;CVD, cardiovascular disease;CAD, coronary artery disease; AF, atrial fibrillation;ACEI, angiotensin-converting enzyme inhibitor; ARB, angiotensin II receptor blocker;K^+^ supp, potassium supplement;GLP-1RA, glucagon-like peptide-1 receptor agonist ;MACE, major adverse cardiovascular events;HR, heart rate;CV, cardiovascular ;HF, heart failure; HHF,hospitalization for heart failure.* represent percentages. | | | | | | | | | | | | | | | | | | | |
